# Recent Advances in Sports Nutrition

**DOI:** 10.1007/s40279-014-0170-1

**Published:** 2014-04-26

**Authors:** L. L. Spriet

**Affiliations:** Department of Human Health and Nutritional Sciences, University of Guelph, Room 354, ANNU Building, Guelph, ON N1G 2W1 Canada

The interest in ‘sports nutrition’ has never been greater. We all know that diet affects athletic performance and that a sound nutritional plan lets an athlete be the best they can be in competition. However, nutrition also plays a large role in the training, adaptation, and preparation for competitions and in the recovery from training and competitions. How well we adapt to, and recover from, one training session or competition often dictates how well we perform in the next training session or competition. The goals of training sessions will also vary in the preparation for competitions. Therefore, sports nutrition is a full-time endeavour in the life of an athlete.

We continually learn more about the importance of nutrition as it relates to sport. Even our basic understanding of the metabolism and interaction of fat and carbohydrate, the major fuels for most forms of exercise, continues to grow. For example, we now know that the regulation of fat metabolism is very complex, with as many sites of regulation as has been demonstrated with carbohydrate metabolism. There has also been increasing interest in the importance of nutrition for the brain in training and competitions, although this information is more difficult to obtain. However, while we advance the knowledge of basic sports nutrition, many practical questions also remain and garner significant research interest.

Athletic performance in many training and competition situations can be optimized with fluid and carbohydrate intake, but are there other nutrients such as protein that could enhance performance, reduce muscle damage, or stimulate protein synthesis? Also, can the adaptations that occur during training be maximized with suboptimal fluid and carbohydrate status or a variety of nutritional approaches? Training-induced adaptations have been studied in traditional organs such as skeletal muscles, but how do other organs, such as the brain and gastrointestinal tract, adapt? In addition, how important are these adaptations for improving athletic performance? Many aspects of these issues are explored in this supplement.

Proper nutrition in the recovery from exercise is also important for maximizing muscular repair, adaptation and hypertrophy, reloading fuel, and improving the ability to sleep and the quality of sleep. If an athlete is well-trained, has a sound nutrition plan, sleeps well, and is healthy, can nutritional supplements such as dietary nitrate or polyphenols help with training, competitions or recovery?

One thing that is very clear is the variability that exists between athletes in almost everything we study. Most coaches, trainers, and sports nutritionists would agree that the increasing amount of information we gather on our athletes leads us to personalize the advice and recommendations we give athletes.

The Gatorade Sports Science Institute (GSSI) brought together researchers for a meeting in April 2012 to discuss many topics of recent interest in the sports nutrition world. Following the meeting, authors were asked to summarize the recent work in their topic, resulting in the manuscripts in this supplement in Sports Medicine.

Lawrence L. Spriet, PhD

Guest Editor

